# Supplementary material for: Human Salivary Histatin-1 Attenuates Osteoarthritis through Promoting M1/M2 Macrophage Transition
Source: Pharmaceutics. 2023 Apr 18;15(4):1272. doi: 10.3390/pharmaceutics15041272 (PMC10147060; doi:10.3390/pharmaceutics15041272)

Supplementary Figure S1

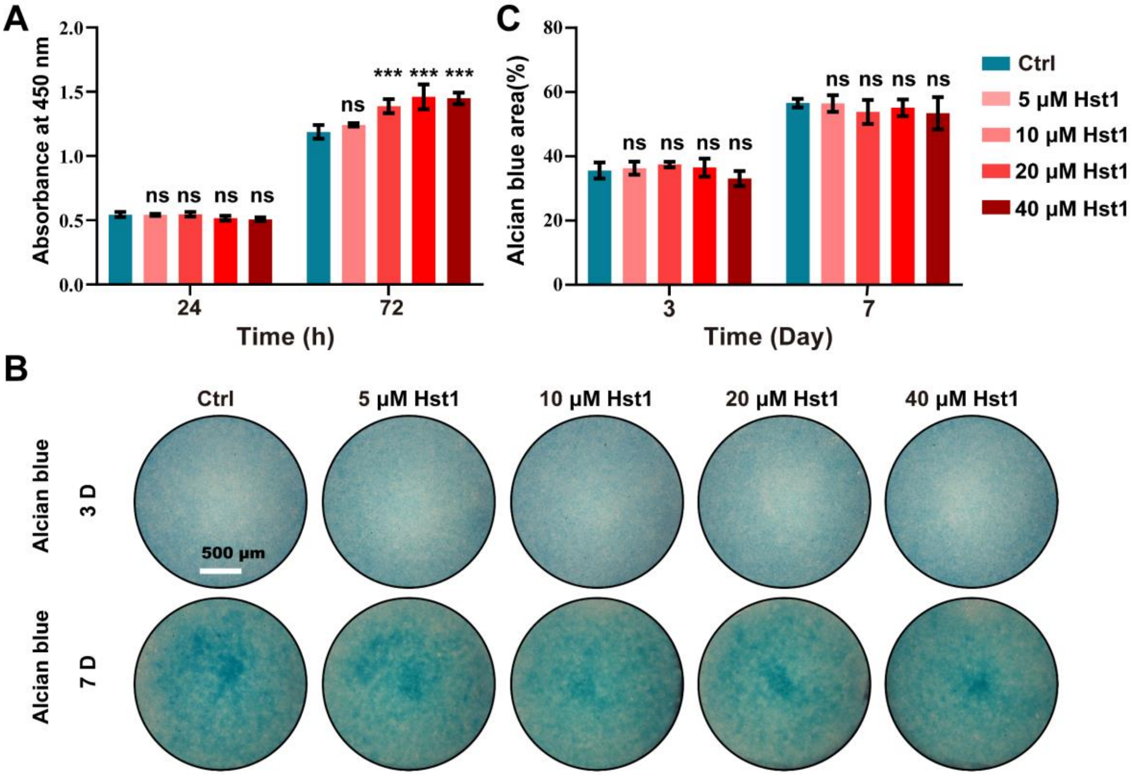

Supplementary Figure S1. Effects of Hst1 on metabolic activity and chondrogenic differentiation of ATDC5 cells. (A) CCK8 assay was used to detecte the effect of Hst1 on the metabolic activity of ATDC5 cells, n=5. (B) and (C) After ATDC5 cells were cultured with Hst1 for 3 and 7 days, Alcian blue was used for staining and quantitative analysis, n=3, scale bar = 500 μm. Data are expressed as mean ± SD. (***: $p < 0.001$ compare with control group. ns = no significant).

files for review purpose

file S1

Uncropped blot images presented in Figure 4E

LPS (100ng/ml)

Hst1 (40 μM) 0 0 40

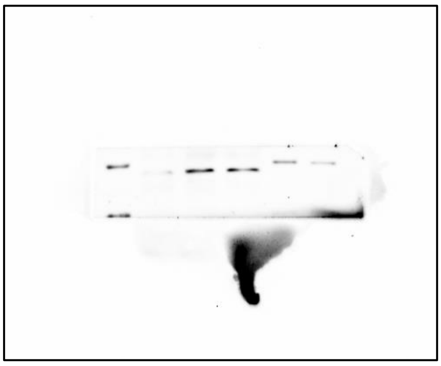

WB (CD86)

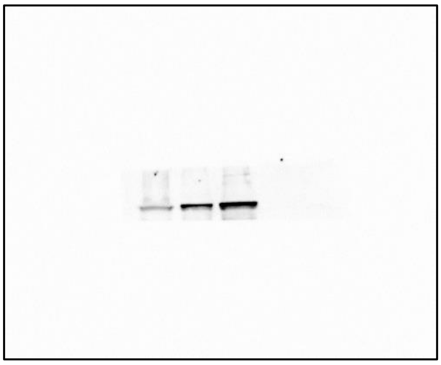

WB (CD206)

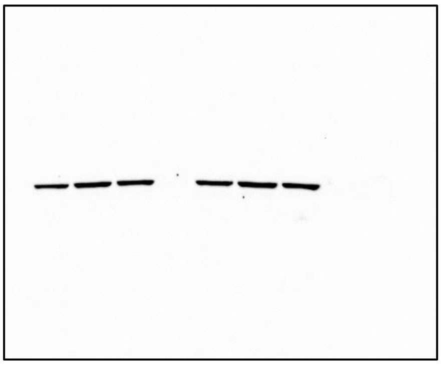

WB (β-actin)

file S2

Uncropped blot images presented in Figure 7A and 7B

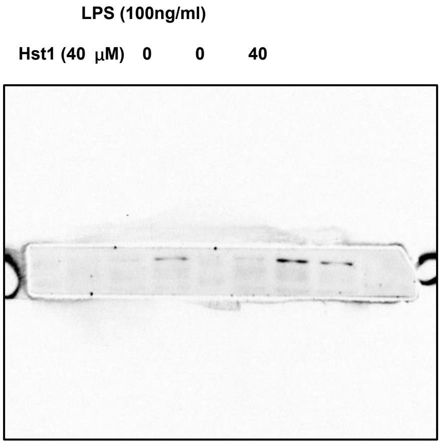

WB (pNF-κB)

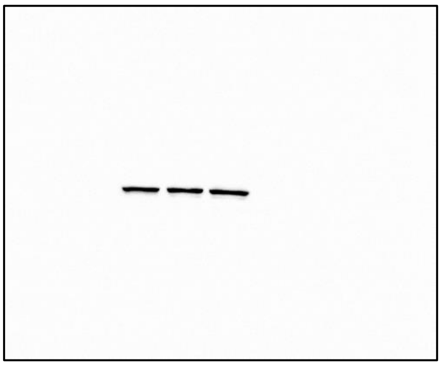

WB (NF-κB)

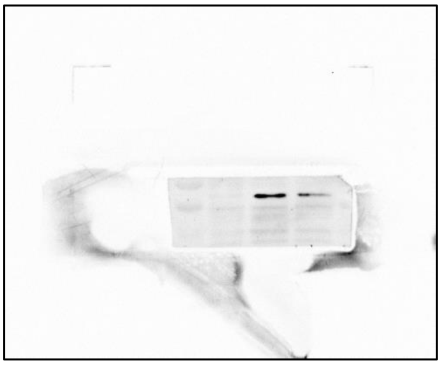

WB (pIKB-α)

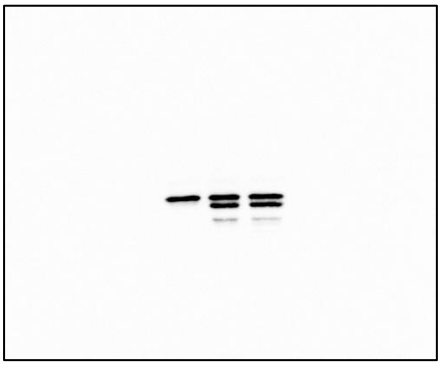

WB (IKB-α)

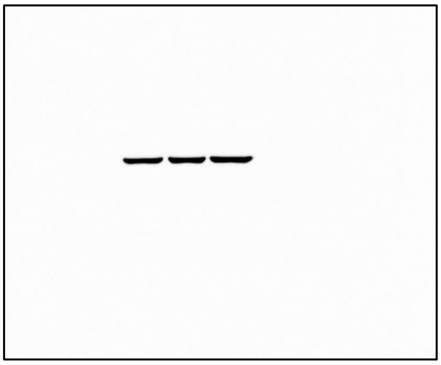

WB (β-actin)

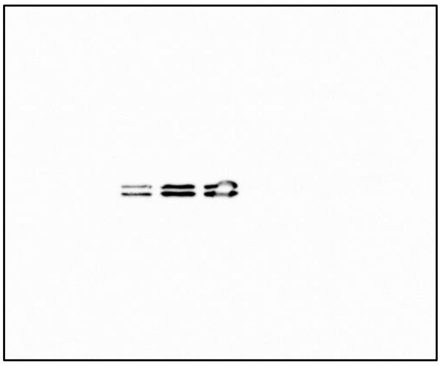

WB (pERK)

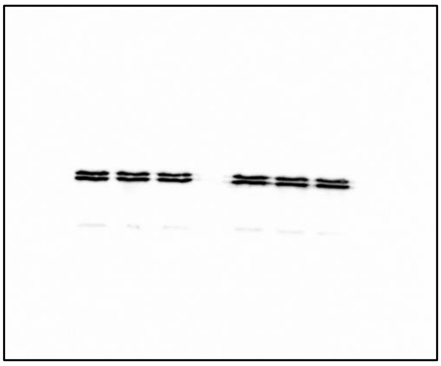

WB (ERK)

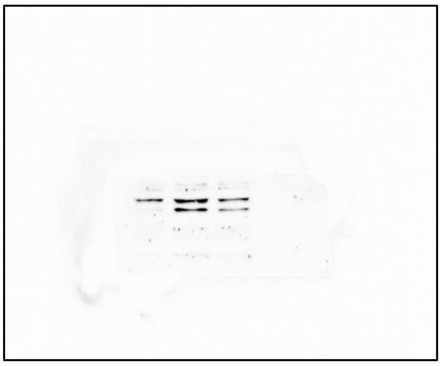

WB (pJNK)

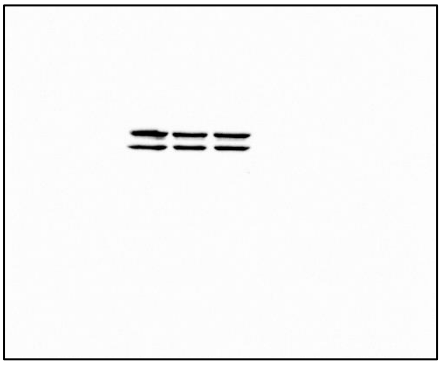

WB (JNK)

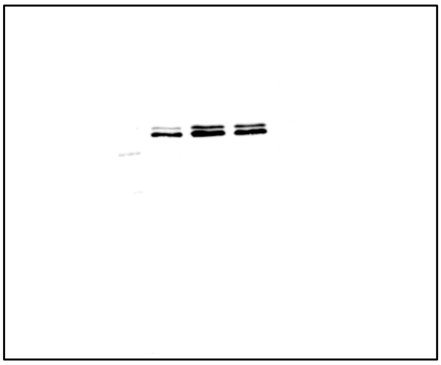

WB (pP38)

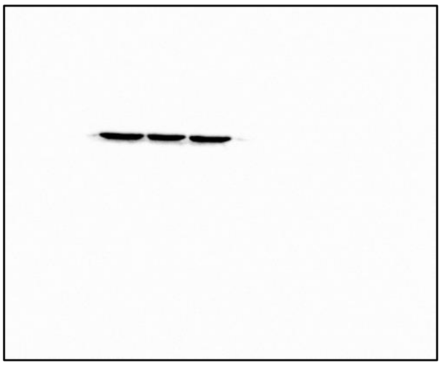

WB (P38)

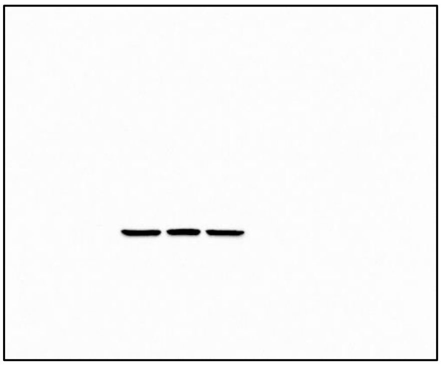

WB (β-actin)

file S3
Uncropped blot images presented in Figure 9B and 9C

Ctrl-CM LPS-CM LPS+Hst1-CM

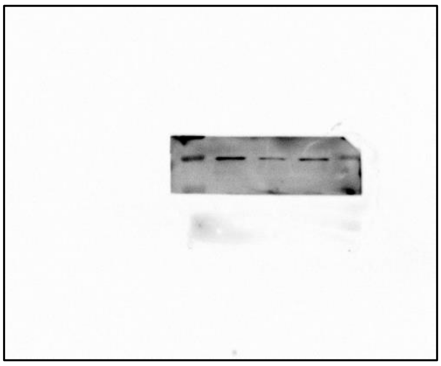

WB (Col-2)

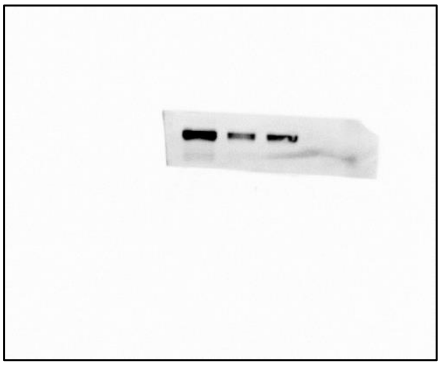

WB (Sox-9)

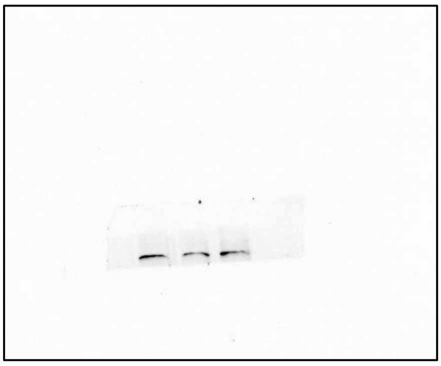

WB (Aggrecan)

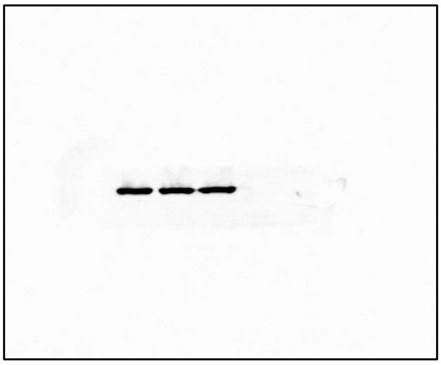

WB (β-actin)

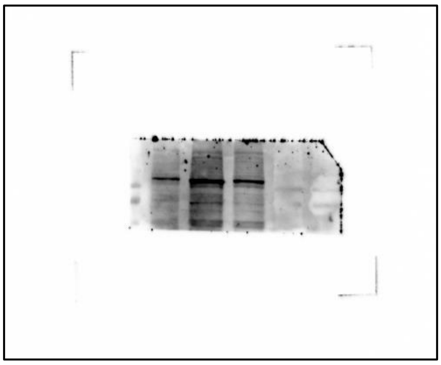

WB (MMP-2)

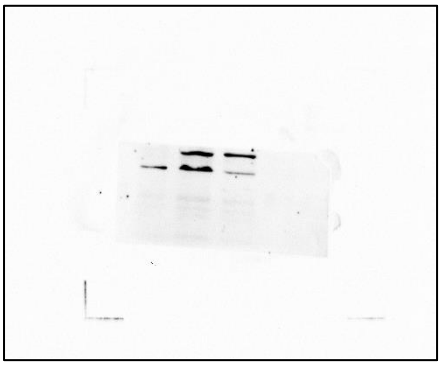

WB (MMP-3)

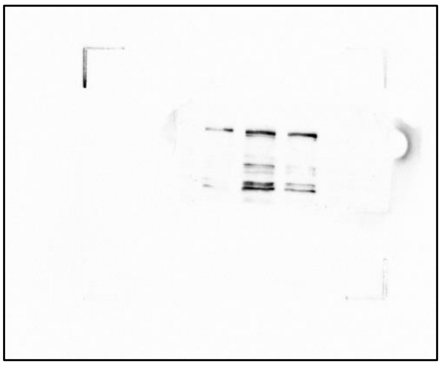

WB (MMP-9)

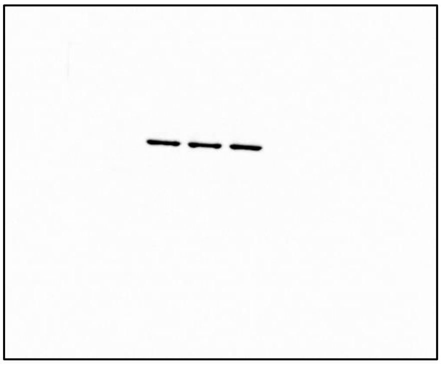

WB (β-actin)

file S4
Uncropped blot images presented in Figure 10 D

Ctrl-CM LPS-CM LPS+Hst1-CM

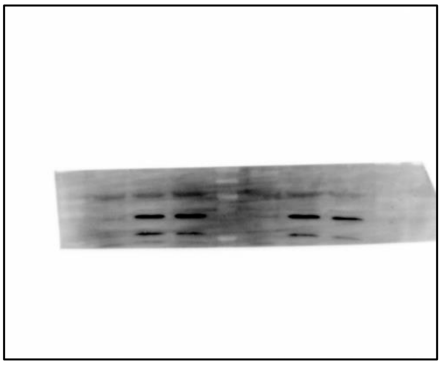

WB (Clv-Caspase3)

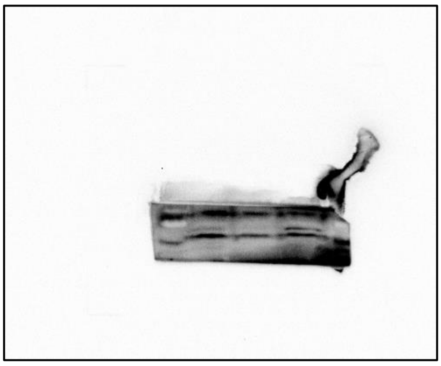

WB (Bcl-2)

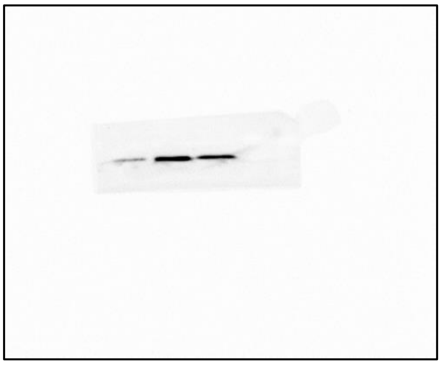

WB (Bax)

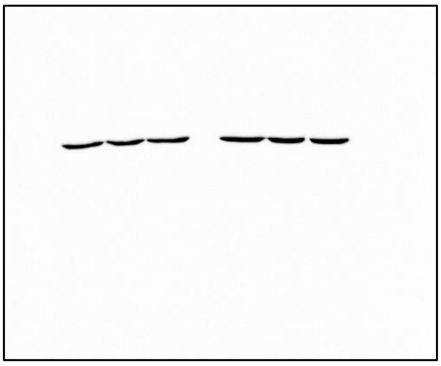

Supplement: Supplementary file 1 [file pharmaceutics-15-01272-s001.zip › pharmaceutics-2251468-supplementary.pdf]
